# Exclusive human milk for infants with gastroschisis: A systematic review and meta-analysis

**DOI:** 10.1177/19345798251384981

**Published:** 2025-10-08

**Authors:** Hareshan Suntharalingam, Jennifer Armstrong, Daniel Briatico, Esther Huisman, Prakesh S Shah, Michael H. Livingston

**Affiliations:** 1 McMaster Pediatric Surgery Research Collaborative, Hamilton, ON, Canada; 2 Department of Health Research Methods, Evidence, and Impact, 3710 McMaster University, Hamilton, ON, Canada; 3 5708 Meharry Medical College, Nashville, TN, USA; 4 Division of Pediatric Surgery, Department of Surgery, 3710 McMaster University, Hamilton, ON, Canada; 5 Division of Neonatology, Department of Pediatrics, 3710 McMaster University, Hamilton, ON, Canada; 6 Division of Neonatology, Department of Pediatrics, 7938 University of Toronto, Toronto, ON, Canada

**Keywords:** breast milk, formula, gastroschisis, necrotizing enterocolitis

## Abstract

**Background:**

Infants with gastroschisis often experience prolonged stays in hospital as they transition from parenteral nutrition to enteral feeds. The purpose of this study was to assess the evidence for the use of exclusive human milk compared to formula in these patients.

**Methods:**

A structured search was performed using Medline, Embase, and Cochrane Central from inception until March 2025. We included comparative studies of infants with gastroschisis who received exclusive human milk versus supplemental or exclusive formula. Screening and data extraction were completed by two independent reviewers. Results from included studies were meta-analyzed using a random-effects model and reported as risk ratios (RR) with 95% confidence intervals (CI). Risk of bias assessments were performed using the Newcastle-Ottawa Scale. Outcomes included time to enteral autonomy, necrotizing enterocolitis, sepsis, cholestasis, and mortality. Certainty of evidence was summarized using Grading of Recommendations, Assessment, Development and Evaluations criteria.

**Results:**

We identified 3661 infants with gastroschisis from six retrospective cohort studies and one case-control study. Exclusive human milk was associated with a reduced risk of mortality (RR = 0.38, 95% CI: 0.15–0.99, I^2^ = 0%). There were no statistically significant differences between groups for necrotizing enterocolitis, cholestasis, or sepsis. These findings were supported by low quality evidence based on retrospective data.

**Conclusions:**

The best available evidence suggests that exclusive breast milk is associated with reduced mortality compared to formula among infants with gastroschisis. This estimate is based on data from retrospective studies. Further research is needed to clarify the role of donor human milk.

## Introduction

Gastroschisis is a congenital abdominal wall defect characterized by the evisceration of intra-abdominal organs. This condition occurs in approximately one in 2400 live births in the United States each year.^1^ Infants with gastroschisis can have simple or complex disease, with the latter associated with intestinal atresia, perforation, necrosis, and/or volvulus. Overall survival is greater than 90% in developed countries but lower than 50% in developing countries, where patients may not have access to advanced neonatal care.^2,3^

After delivery, infants with gastroschisis undergo abdominal closure via “sutureless” or primary fascial techniques, or in a delayed fashion with placement of a temporary silo. Patients are supported with parenteral nutrition during the postnatal period due to impaired intestinal function. As a result, infants with gastroschisis are susceptible to impaired growth.^4–6^

Early commencement of enteral feeds among infants with gastroschisis has been shown to shorten length of stay, days on parenteral nutrition, and time to full enteral feeds.^7^ This practice also facilitates weight gain and reduces the risk of parenteral nutrition-associated cholestatic liver disease and sepsis.^8^ The benefits of early enteral feeding, however, must be balanced against the risk of feeding intolerance and necrotizing enterocolitis.

Most centers encourage the use of human milk for infants with gastroschisis. According to the World Health Organization, human milk is essential for growth and development as it contains immunoglobulins, cytokines, growth factors, essential fatty acids, lactoferrin, amino acids, and human milk oligosaccharides.^9,10^ Human milk is also an excellent source of probiotics, which support the colonization of commensal gut bacteria.^11^ Human milk oligosaccharides nourish gut microflora, prevent binding of pathogenic microbes, and maintain the integrity of the intestinal barrier.^11,12^ Byproducts of the microbial fermentation of human milk oligosaccharides also play a crucial role in the maturation of intestinal epithelial cells.^12^

Human milk can be categorized as either mother’s own milk or donor human milk. Mother’s own milk refers to breast milk expressed by the infant’s own biological mother and typically serves as the primary source of nutrition for the newborn.^13^ However, donor human milk is required if the maternal supply is insufficient or if there are any contraindications, such as mothers with blood-borne infections (e.g., human immunodeficiency virus, human T-cell leukemia-lymphoma virus, or cytomegalovirus).^13^

A systematic review by Quigley et al. showed that human milk significantly reduced the risk of necrotizing enterocolitis in preterm and very low birth weight infants.^14^ To the best of our knowledge, however, there is no systematic review on the effects of exclusive human milk among infants with gastroschisis.

The purpose of this study was to systematically review studies of exclusive human milk compared to supplemental or exclusive formula among infants with gastroschisis. Our research question was: “Among infants with gastroschisis, is exclusive human milk feeding associated with improved outcomes compared to supplemental or exclusive formula feeding?”

## Methods

### Study design

This systematic review was completed in accordance with the Meta-analyses of Observational Studies in Epidemiology (MOOSE) statement and Preferred Reporting Items for Systematic Review and Meta-Analysis (PRISMA) guidelines.^15,16^ The protocol was registered in PROSPERO on June 10, 2023 (CRD42023426801).^17^

### Search strategy

A systematic search was performed using Medline, Embase, and Cochrane Central Register of Controlled Trials from inception to March 6, 2025, for any relevant articles that met the predefined eligibility criteria. The search strategy for each database can be found in the Appendix 1–3. Additionally, we reviewed trial registries,^18,19^ and reference lists of included studies. A manual search was also conducted to capture any other relevant studies that were missed.

### Eligibility criteria

We considered both randomized controlled trials and comparative observational studies on the effectiveness of exclusive human milk versus supplemental or exclusive formula among infants with gastroschisis. Case reports, case-series, articles based on expert opinion, conference abstracts, reviews, and studies published in languages other than English were excluded. Studies that included infants with gastrointestinal conditions other than gastroschisis were excluded in order to maintain a homogenous study population. Gastroschisis is a condition with significant heterogeneity, and we wanted to avoid any potential confounding from other gastrointestinal conditions (e.g., intestinal atresia).

Studies that reported data on any of the following outcomes of interest were included: (1) time to full enteral autonomy (i.e., time from commencement of enteral feeds to discontinuation of parenteral nutrition); (2) duration of parenteral nutrition; (3) length of stay; (4) infant weight at discharge; (5) incidence of necrotizing enterocolitis (of any stage); (6) sepsis (i.e., laboratory-confirmed bloodstream infection); (7) cholestatic liver disease (i.e., laboratory evidence of cholestasis); and (8) mortality.

### Study selection

The records identified in our search were imported to Covidence. Two reviewers (HS and JA) completed title and abstract screening independently. In the event of a conflict, the record was automatically included for full-text screening. Included records were uploaded to Covidence, where full-text screening was performed independently and in duplicate. Any disagreements were resolved by the senior author (MHL).

Data extraction was carried out using a piloted form on Covidence. The two reviewers (HS and JA) were blinded to each other’s responses. Extracted data included study characteristics (e.g., country, years of recruitment, and number of participants), baseline characteristics (e.g., gastroschisis severity), and outcomes.

### Risk of bias

Two reviewers (HS and JA) independently completed risk of bias assessments using the Newcastle-Ottawa Scale (NOS) for non-randomized studies.^20^ The NOS is appropriate for assessing risk of bias in cohort and case-control studies. This instrument is composed of three domains that assess selection bias, comparability of treatment arms, and bias in either outcome ascertainment (for cohort studies) or exposure ascertainment (for case-control studies). Total scores can range from 0 to 9 stars, where studies with scores of 7–9, 4–6, and <4 stars are considered to represent low, moderate, and high risk of bias, respectively.^21,22^

Funnel plots were created using Review Manager to assess possible publication bias. Asymmetry in the plots was determined using visual inspection.

### Certainty of evidence

The certainty of evidence was assessed using Grading of Recommendations, Assessment, Development and Evaluation (GRADE).^23^ The GRADE rating is a composite evaluation of the included studies for a given outcome based on risk of bias, inconsistency, indirectness, imprecision, and publication bias. Outcomes from non-randomized studies were initially assigned a rating of low certainty. However, the ratings were upgraded if there was evidence of a dose-response relationship, large effect size, and/or plausible opposing residual confounding.

### Statistical analysis

Dichotomous variables were reported as proportions and continuous variables were reported using means and standard deviations. Whenever possible, data were meta-analyzed using Review Manager 7.2.0. Risk ratios (RR) and 95% confidence intervals (CI) were calculated for the dichotomous outcomes. Forest plots were generated using a random-effects model as we anticipated variation between studies due to differing practice patterns between neonatal intensive care units. Weights were assigned for the individual studies using the Mantel-Haenszel method. We planned to conduct a subgroup analysis if feasible to assess the impact of complex gastroschisis. Narrative syntheses were conducted for outcomes with insufficient data for meta-analysis.

Heterogeneity was evaluated using I^2^ statistics and chi-square tests, with a p-value threshold of less than 0.05. I^2^ values between 0% and 40% are deemed to be of minimal importance, 30%–60% may represent moderate heterogeneity, 50%–90% may represent substantial heterogeneity, and 75%–100% represents considerable heterogeneity.^24^

## Results

### Study selection

We identified 197 records, of which 43 duplicates were removed and the remaining 154 underwent title and abstract screening. Twenty-seven were selected for full-text screening. The most common reason for exclusion after full-text screening was that the interventions studied were not relevant to the primary research question. Studies were excluded because they assessed the impact of multifaceted quality improvement initiatives or clinical care pathways,^25–29^ use of colostrum,^30^ sham feeding,^31^ oral immune therapy,^32^ gestational age,^33^ dose-response relationship involving human milk,^34^ or the supplementation of mother’s own milk with donor human milk.^35^

Four studies were excluded because they reported outcomes for multiple gastrointestinal conditions in aggregate rather than gastroschisis separately.^36–39^ Two other studies were excluded because they focused on outcomes that were not relevant to the primary research question.^40,41^ One of these compared the nutritional profiles of infants with simple gastroschisis to their counterparts with complex gastroschisis,^40^ while another investigated the effects of human milk on the gut microbiome.^41^ A literature review featuring a case report,^42^ and a conference abstract were also excluded.^43^

**Figure 1.**
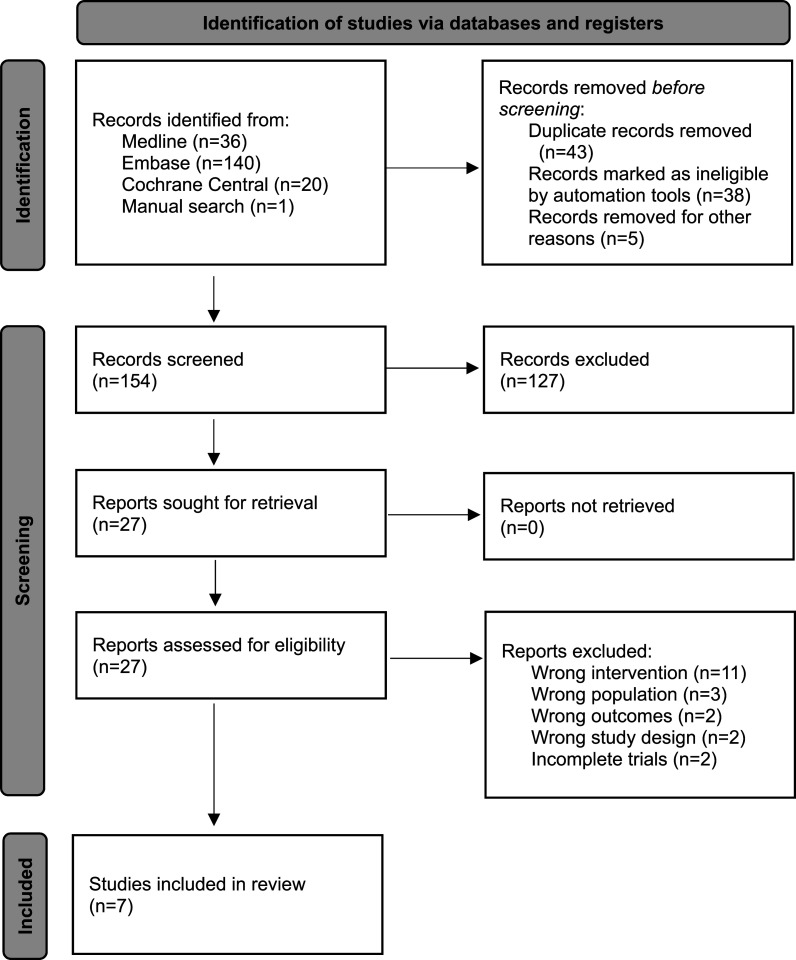
PRISMA flow diagram outlining the selection of studies.

We identified one single-center, randomized controlled trial comparing exclusive human milk with formula.^44^ This study was terminated due to low participant recruitment after enrollment of four participants. Seven observational studies were selected for inclusion.^8,45–50^ The PRISMA flow diagram summarizing study selection is shown in Figure 1.

### Study characteristics

Our search strategy did not identify any randomized controlled trials that completed enrollment. The seven studies selected for inclusion consisted of six retrospective cohort studies and one case-control study. This resulted in a total sample of 3661 infants with gastroschisis.

Three of the included studies were conducted in the United States,^45–47^ two in Canada,^48,49^ one in Australia,^8^ and one in the United Kingdom.^50^ Four studies restricted their inclusion criteria to infants with simple gastroschisis.^8,47–49^ Two studies excluded infants with very low birthweight (i.e., less than 1500 g).^48,49^

The mean gestational age was approximately 36 weeks in five of the seven studies.^8,46,48–50^ Two studies excluded infants who were born very premature (i.e., <32 weeks).^48,49^ In another study, 65% of participants were premature.^45^ The characteristics of the included studies are summarized in Table 1.

**Table 1.** Characteristics of included studies.

Source	Study design	Sample size (*n*)	Country	Years recruited	Gastroschisis severity	Silo closure	Gestational age (weeks)^a^	Birthweight (grams)^a^
Hodgson (2024)	Retrospective cohort (multicenter)	267	Canada	2014–2022	Simple	77 (29%)	36 (1.2)	2499 (478)
Hodgson (2022)	Retrospective cohort (single center)	75	Canada	2005–2019	Simple	41 (55%)	35.9 (1.4)	2507 (407)
Storm (2020)	Retrospective cohort (single center)	44	United States	2005–2016	Simple and complex	Not reported	35.7 (33.9, 37.1)^b^	2189 (577)
Thompson (2017)	Retrospective cohort (single center)	60	Australia	2010–2016	Simple	23 (38%)	36 (30–39)^c^	2400 (1300–3860)^c^
Gulack (2016)	Retrospective cohort (multicenter)	3082	United States	1997–2012	Simple and complex	Not reported	Not reported	Not reported
Kohler (2013)	Retrospective cohort (single center)	79	United States	2000–2010	Simple	42 (53%)	Not reported	Not reported
Jayanthi (1998)	Case-control study (single center)	54	England	1990–1996	Not reported	12 (22%)	36.4 (2.1)	2331 (505)

^a^Mean (standard deviation) unless otherwise stated.

^b^Median (interquartile range).

^c^Median (range).

### Risk of bias

Assessments with the NOS suggested that the risk of bias ranged from low to moderate across the seven included studies, with scores ranging from 5 to 8 stars. In the selection domain, one star was deducted for representativeness of the exposed cohort for four studies, as patients were recruited over a 10-year period.^45–48^ Such an extended recruitment timeframe may have introduced variability in patient management due to changes in clinical practice, such as the adoption of the sutureless closure technique, earlier initiation of enteral feeding, and advances in neonatal care.

One star was deducted from the comparability domain for two studies since they did not control for the complexity of gastroschisis.^45,50^ Another star was deducted in two studies because they did not control for any other clinically important prognostic variables.^8,50^ A star was not awarded for exposure ascertainment under the selection domain for two studies because the data were retrieved primarily from registries or databases.^45,49^ An additional star was deducted from the outcome domain due to inadequate length of follow-up.^45,49^ The NOS scores are summarized in Table 2.

**Table 2.** Risk of bias of individual studies using Newcastle-Ottawa Scale.

Study	Selection	Comparability	Outcome/exposure	Overall
Hodgson (2024)	★★★	★★	★★	7/9
Hodgson (2022)	★★★	★★	★★★	8/9
Storm (2020)	★★★	★★	★★★	8/9
Thompson (2017)	★★★★	★	★★★	8/9
Gulack (2016)	★★	★	★★	5/9
Kohler (2013)	★★★	★★	★★★	8/9
Jayanthi (1998)	★★★★		★★★	7/9

Publication bias was assessed using funnel plots for the clinical outcomes that were meta-analyzed (Appendixes 4–7). There did not appear to be definitive evidence of publication bias but the number of studies that were included in each funnel plot was small.

### Outcomes

The studies selected for inclusion reported a range of clinical outcomes. Dichotomous outcomes included necrotizing enterocolitis, sepsis, cholestasis, reoperation, readmission, and mortality. Time-to-event outcomes included length of stay, time to discharge from first feed, and time to full enteral feeds. An overview of the outcomes reported in each study is included in Table 3.

**Table 3.** Outcomes reported by included studies.

Outcome	Hodgson (2024)	Hodgson (2022)	Storm (2020)	Thompson (2017)	Gulack (2016)	Kohler (2013)	Jayanthi (1998)
Necrotizing enterocolitis	Yes	Yes	—	—	Yes	—	Yes
Cholestasis	Yes	Yes	—	—	—	—	Yes
Sepsis	Yes	Yes	—	—	Yes	—	—
Mortality	Yes	Yes	—	—	Yes	—	—
Time to full enteral feeds	Yes	Yes	—	—	—	Yes	—
Length of stay	Yes	Yes	Yes	Yes	—	Yes	—
Duration of parenteral nutrition	Yes	Yes	Yes	Yes	—	—	—
Time to discharge from first feed	—	—	Yes	—	Yes	Yes	—

### Necrotizing enterocolitis

**Figure 2.**
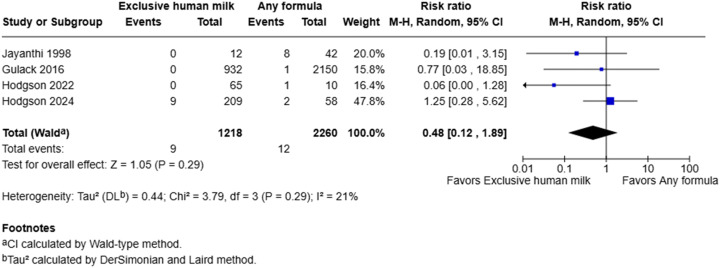
Forest plot of necrotizing enterocolitis among infants with gastroschisis who received exclusive human milk compared to supplemental or exclusive formula.

Three retrospective cohort studies and one case-control study reported the incidence of necrotizing enterocolitis.^45,48–50^ Three of these studies suggested that exclusive human milk was associated with decreased risk of necrotizing enterocolitis.^45,48,50^ The other study demonstrated similar rates among those who received exclusive human milk compared to those who receive supplemental or exclusive formula.^49^ Meta-analysis suggested that exclusive human milk was not definitively associated with risk of necrotizing enterocolitis (RR = 0.48, 95% CI: 0.12–1.89, I^2^ = 21%, *p* = 0.29) (Figure 2).

### Cholestasis

**Figure 3.**
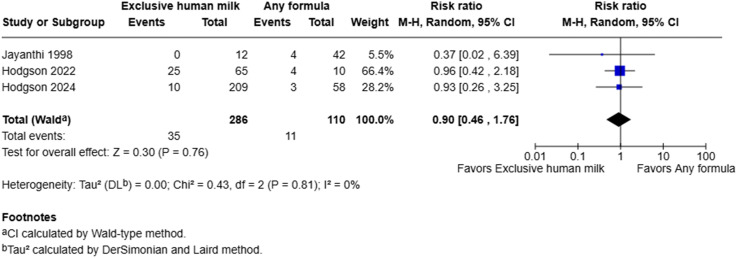
Forest plot of cholestasis among infants with gastroschisis who received exclusive human milk compared to supplemental or exclusive formula.

The frequency of cholestasis was reported in three studies.^48–50^ Two of which found no difference in the risk of developing cholestasis between treatment groups.^48,49^ In contrast, the case-control study by Jayanthi et al. suggested that exclusive human milk was associated with a lower risk of cholestasis (0% vs 9.5%).^50^ Our own post hoc analysis with a one-tailed Fisher’s exact test suggested that this difference was not statistically significant (*p* = 0.35). This study also included mild cases in their definition of cholestasis. The pooled estimate from the meta-analysis suggested that exclusive human milk was not associated with risk of cholestasis (RR = 0.90, 95% CI: 0.46–1.76, I^2^ = 0%, *p* = 0.76) (Figure 3).

### Sepsis

The incidence of sepsis was reported in three studies.^45,48,49^ In the study by Gulack et al., the incidence of sepsis was reported as “positive blood culture after first feed.” This outcome was nearly four times lower among infants who were fed exclusive human milk compared to those who received supplementary or exclusive formula (3.2% vs 11.7%, *p* < 0.01).^45^ Conversely, the multicenter study by Hodgson et al. found that infants in the exclusive human milk group were twice as likely as the formula-fed group to develop sepsis.^49^ The single-center study by Hodgson et al. demonstrated that the rates of sepsis were similar between both groups.^48^

**Figure 4.**
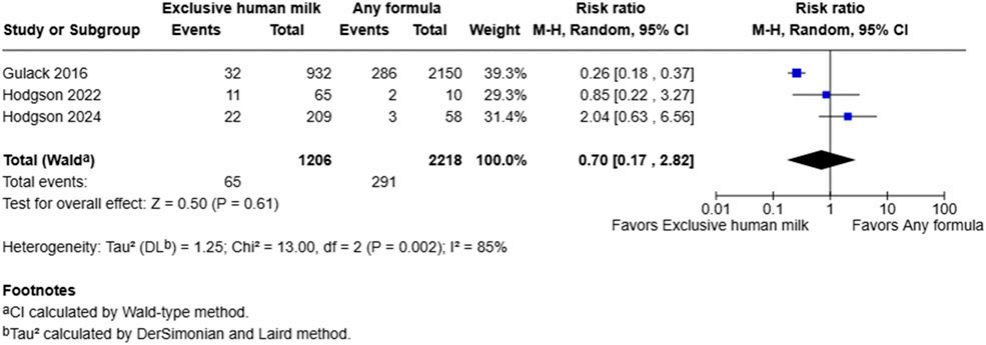
Forest plot of sepsis among infants with gastroschisis who received exclusive human milk compared to supplemental or exclusive formula.

Meta-analysis demonstrated that administration of exclusive human milk was not definitively associated with the risk of sepsis (RR = 0.70, 95% CI: 0.17–2.82, I^2^ = 85%, *p* = 0.61) (Figure 4). However, there was substantial heterogeneity between studies, as indicated by the high I^2^ statistic. Only one of the three studies clearly reported tracking infection rates after the initiation of enteral feeds.^45^ Hence, the infection rates in the other two studies may also include infections that occurred prior to the commencement of enteral feeds. Another potential source of heterogeneity may be the substantially larger sample size in the study by Gulack et al. compared to the other two studies by Hodgson et al. As a result, the latter two studies may be underpowered to accurately capture the effects of exclusive human milk on the risk of sepsis, given that their primary outcome of interest was time to full enteral feeds.

### Mortality

Three cohort studies reported the incidence of mortality.^45,48,49^ There was a slightly elevated risk of mortality in the exclusive human milk group in the two studies by Hodgson et al.^48,49^ However, there was only one documented case of mortality in each of the two studies. Furthermore, the number of participants who received exclusive human milk was more than twice that of the formula group in both studies. In contrast, the study by Gulack et al. demonstrated that exclusive human milk can significantly reduce the risk of mortality.^45^ However, the authors did not adjust for differences between groups in terms of the severity of gastroschisis at baseline.

**Figure 5.**
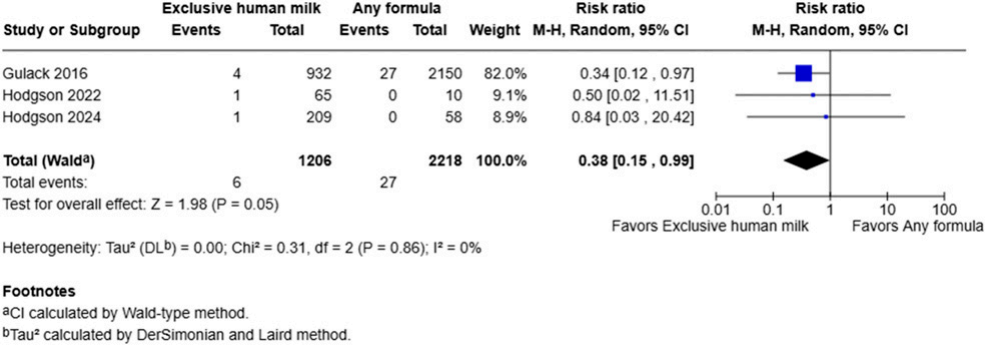
Forest plot of mortality among infants with gastroschisis who received exclusive human milk compared to supplemental or exclusive formula.

Meta-analysis suggested that the use of exclusive human milk was associated with a statistically significant reduction in the risk of mortality compared to supplemental or exclusive formula (0.5% vs 1.2%) (RR = 0.38, 95% CI: 0.15 to 0.99, I^2^ = 0%, *p* = 0.05) (Figure 5). However, the number of deaths was very low, especially in the two studies by Hodgson et al. This limited the precision of the estimate and resulted in wide confidence intervals.

### Time to full enteral feeds

Time to full enteral feeds was reported in three studies.^47–49^ This outcome was defined as the time from the commencement of enteral nutrition to the cessation of parenteral nutrition in the single-center study by Hodgson et al.^48^ Similarly, the study by Kohler et al. used the start of enteral feeding as the initial time point,^47^ however, they operationalized the end point as the ability to tolerate enteral feeds of 140 ± 10 ml/kg/day.

In the multicenter study by Hodgson et al.,^49^ exclusive human milk was associated with a delay in achieving enteral autonomy (median 24 vs 22 days, *p* = 0.047). However, there was no difference in the time to achieve enteral autonomy after adjusting for sex and timing of abdominal closure. Kohler et al. found that exclusive human milk was associated with decreased time to full enteral feeds (median five vs 7 days, *p* = 0.03).^47^ This was consistent with the findings of the single-center study by Hodgson et al. (median 18 vs 25 days, *p* = 0.023).^48^ Data for time to full enteral feeds were not meta-analyzed due to differences in reporting between studies.

### Length of stay

Five of seven studies reported length of stay.^8,46–49^ In the multicenter study by Hodgson et al., length of stay was shorter by 1 day among infants who were received exclusive human milk compared to infants who received any amount of formula (median 28 vs 29 days, *p* = 0.93).^49^ The single-center study by Hodgson et al. demonstrated a trend toward shorter length of stay among infants in the exclusive human milk group (median 27 vs 46 days, *p* = 0.057).^48^ The study by Kohler et al. also showed a reduction in length of stay (median 22 vs 30 days, *p* = 0.001).^47^

Storm et al. demonstrated a dose-response relationship between the administration of human milk and shorter hospitalization periods using Cox proportional hazards modeling. In this study, the adjusted hazard ratio (HR) for discharge per 10% increase in mother’s own milk dose was 1.130 (95% CI: 1.028–1.242, *p* = 0.011).^46^ A stronger association was observed when comparing infants who received exclusive human milk with those who received exclusive or supplemental formula (adjusted HR: 6.365, 95% CI: 2.072–19.547, *p* = 0.001).^46^ Thompson et al. did not report any difference in length of stay between both groups.^8^ Data for length of stay were not meta-analyzed due to differences in the format of results between studies.

### Other outcomes

The duration of parenteral nutrition was reported in four studies.^8,46,48,49^ Duration on parenteral nutrition was used as a surrogate measure for time to full feeds in the study by Thompson et al.^8^ There were no differences in three of four studies.^8,46,49^ One study found a shorter duration of parenteral nutrition among infants who received exclusive human milk (median 20 vs 26 days, *p* = 0.037).^48^ All three studies which assessed the time to discharge from first enteral feed found a consistently shorter time to discharge among infants who received exclusive human milk.^45–47^ Notably, Gulack et al. found a statistically significant reduction in the time to discharge among infants with gastroschisis who were fed exclusive human milk compared to those who received any formula (*p* < 0.01).^45^ This finding is particularly meaningful given the substantially large sample size of over 3000 participants.

### Certainty of evidence

The certainty of evidence according to GRADE criteria was very low across the four dichotomous outcomes that were meta-analyzed (Table 4). Ratings were downgraded for necrotizing enterocolitis, cholestasis, sepsis, and mortality due to imprecision, which resulted from a low number of events and/or 95% CIs that crossed the line of no difference. The quality of evidence for sepsis was further downgraded due to inconsistency, given the high heterogeneity between studies (I^2^ = 85%). The ratings for sepsis and mortality were also reduced due to the inclusion of a study with considerable weight that was found to be at a moderate risk of bias. However, certainty ratings were upgraded for necrotizing enterocolitis and mortality due to the large estimates of treatment effect (i.e., RR >2 or <0.5).

**Table 4.** Grading of recommendations, assessment, development, and evaluation (GRADE) summary of evidence.

Certainty assessment							Number of patients		Effect		Certainty	Importance
Number of studies	Study design	Risk of bias	Inconsistency	Indirectness	Imprecision	Other considerations	Exclusive human milk	Any formula	Relative (95% CI)	Absolute (95% CI)		
Necrotizing enterocolitis												
4	Non-randomized studies	Not serious	Not serious	Not serious	Very serious^a,b^	Strong association^c^	9/1218 (0.7%)	12/2260 (0.5%)	RR 0.46 (0.11 to 1.95)	3 fewer per 1000 (from 5 fewer to 5 more)	⨁◯◯◯	Important
											Very low^a,b,c^	
Cholestasis												
3	Non-randomized studies	Not serious	Not serious	Not serious	Serious^a,b^	None	35/286 (12.2%)	11/110 (10.0%)	RR 0.90 (0.46 to 1.76)	10 fewer per 1000 (from 54 fewer to 76 more)	⨁◯◯◯	Important
											Very low^a,b^	
Sepsis												
3	Non-randomized studies	Serious^d^	Very serious^e^	Not serious	Serious^a^	None	65/1206 (5.4%)	291/2218 (13.1%)	RR 0.70 (0.17 to 2.82)	39 fewer per 1000 (from 109 fewer to 239 more)	⨁◯◯◯	Important
											Very low^a,d,e^	
Mortality												
3	Non-randomized studies	Serious^d^	Not serious	Not serious	Serious^b^	Strong association^c^	6/1206 (0.5%)	27/2218 (1.2%)	RR 0.38 (0.15 to 0.99)	8 fewer per 1000 (from 10 fewer to 0 fewer)	⨁◯◯◯	Critical
											Very low^b,c,^^d^	

CI: confidence interval; RR: risk ratio.

^a^95% confidence intervals span risk ratio of 1.0.

^b^Low number of events (<300 events).

^c^Large pooled effect estimate (i.e., RR >2 or <0.5).

^d^See Table 2.

^e^Substantial variability between studies (i.e., I^2^>40%).

## Discussion

This systematic review included seven retrospective studies of 3661 infants with gastroschisis. Our meta-analysis demonstrated that there is very low certainty evidence supporting an association between the use of exclusive breast milk and reduced mortality. Very low certainty evidence indicated no definitive association between exclusive human milk and necrotizing enterocolitis, sepsis, or cholestasis. The infants included in this systematic review were more likely to receive supplementary or exclusive formula compared to exclusive human milk. The limited availability of mother’s own milk may be the result of delays in being able to start breastfeeding. This challenge is seen with other congenital gastrointestinal anomalies, such as congenital diaphragmatic hernia, duodenal atresia, and esophageal atresia.^51^ Unless mothers proactively pump and store human milk in the postnatal period, infants are dependent on donor human milk or formula when enteral feeds are started.

Hodgson et al. demonstrated an association between exclusive breastfeeding at discharge and the provision of exclusive human milk within the first 28 days of life.^49^ This finding underscored the importance of the timely introduction of non-nutritive feeding to encourage the development of oral motor skills and breastfeeding during the later stages of infancy. Additionally, they found that maternal sociodemographic factors such as age and history of substance use did not play a role in whether their infants received human milk or formula.^49^

Necrotizing enterocolitis has been reported to affect up to 20% of infants with gastroschisis,^50^ and is predictive of sepsis and mortality.^52^ Our meta-analysis demonstrated that exclusive human milk may or may not confer protection against necrotizing enterocolitis among infants with gastroschisis, since this trend was not statistically significant. However, a meta-analysis of randomized controlled trials comparing human milk versus formula in low birthweight infants demonstrated that exclusive human milk was associated with a decreased risk of necrotizing enterocolitis.^53^ Similar findings were reported in another systematic review, where a higher dosage of human milk was associated with reduced risk of necrotizing enterocolitis in very low birthweight infants.^54^

Our narrative synthesis suggested that the exclusive human milk may be associated with a shorter time to enteral autonomy. This physiological response can be explained by the faster gastric emptying observed when infants are fed human milk compared to those who received formula (mean 47 vs 64 min; *p* < 0.05).^55^ Infants with gastroschisis often experience intestinal dysmotility following abdominal closure. As a result, they are prone to delayed gastric emptying and gastroesophageal reflux, both of which can impair their ability to tolerate enteral feeds.^56^ Furthermore, three of the studies included in this review reported that exclusive human milk was associated with decreased time to discharge from initiation of enteral feeds.^45–47^

In addition to nourishing newborns with the necessary macronutrients, mother’s own milk confers immunological protection due to the presence of of leukocytes and a host of immunoglobulins, including IgA, IgG, and IgM.^57,58^ Our meta-analysis suggested that exclusive human milk may or may not affect the risk of sepsis, since there was substantial heterogeneity between studies. This differs from a systematic review of breastfeeding in healthy term infants, which reported that human milk offers protection against gastrointestinal and respiratory infections.^59^

None of the studies included in this review assessed the use of donor human milk among infants with gastroschisis. Pasteurization of donor human milk is critical to remove pathogens but can also lower concentrations of immunoglobulins, lymphocytes, lipoprotein lipase, bile salt-dependent lipase, and the activity of proteins that are implicated in the immune response.^60,61^ We recently conducted a survey of feeding practices for infants with gastroschisis among neonatal intensive care units across Canada and found variation in the use of donor human milk in these patients.^62^

A growing body of evidence suggests that the use of standardized feeding protocols for infants with gastroschisis is associated with a lower incidence of sepsis, length of stay, and time to full feeds.^63–66^ Feeding protocols may modify the effect of exclusive human milk by optimizing critical aspects of neonatal nutrition, including the initiation of trophic feeds, early commencement of nutritive feeds, and gradual advancement to minimize the risk of feeding intolerance and necrotizing enterocolitis.^63^ As a result, feeding protocols may enhance the benefits associated with exclusive human milk. At least one neonatal intensive care unit in Canada is in the process of developing a feeding protocol for infants with gastroschisis.^62^ We are hopeful that others will do the same and encourage the use of exclusive human milk whenever possible.

This study has several limitations. First, none of the studies included in this review assessed outcomes beyond the index hospital admission, such as those related to the gut microbiome, immune function, growth, and neurodevelopmental outcomes. Follow-up assessments for neurodevelopmental abnormalities (e.g. Bayley scales of infant and toddler development) were not feasible as the retrospective data from the included studies were collected during the index hospital admission. As a result, the long-term effects of exclusive human milk among infants with gastroschisis remain uncertain. We were also unable to perform subgroup analyses based on disease complexity, since the included studies presented aggregate data for infants with both uncomplicated and complicated disease, or explicitly excluded infants with complicated gastroschisis. Finally, our systematic search was limited to articles published in English. This may have introduced language bias and impacted the generalizability of the findings.

The certainty of evidence according to GRADE criteria was very low across all outcomes despite a sufficiently large sample size. This was because the included studies were observational and had problems with imprecision and inconsistency. Certainty ratings were upgraded for necrotizing enterocolitis and mortality due to the large estimates of treatment effect. However, the magnitude of pooled estimates may change as additional data become available.

Conducting randomized controlled trials on rare and heterogenous conditions such as gastroschisis is challenging. We identified at least one center that attempted to conduct a randomized controlled trial comparing exclusive human milk and formula among infants with gastroschisis.^44^ Unfortunately, this study had to be stopped early due to poor recruitment. Beyond the logistical difficulties of recruiting a sufficiently large sample for a trial on a rare condition like gastroschisis, there are also ethical concerns. The current evidence suggests that exclusive breast milk is likely superior to formula for infants with gastroschisis. As such, there is no clinical equipoise between these two treatment options, and a randomized controlled trial is not justified. Future research efforts should be directed toward developing institutional care pathways and clinical practice guidelines to establish best practices for infants with gastroschisis. This is important to standardize clinical care and work toward achieving the best possible patient outcomes.

## Figures and Tables

**Appendix 1.**
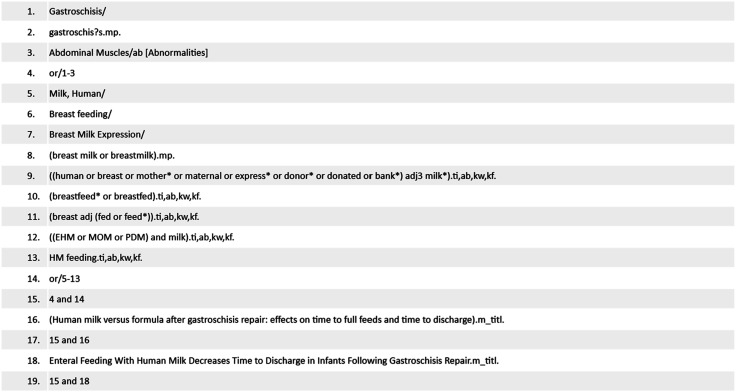
Search strategy for Medline.

**Appendix 2.**
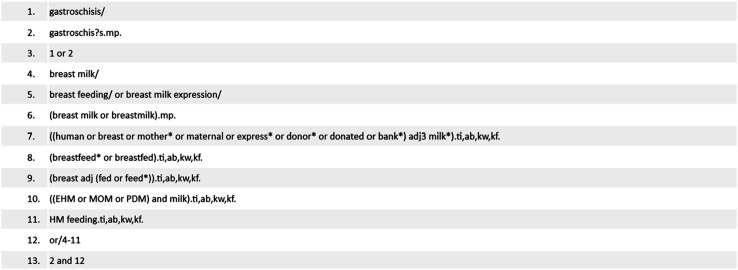
Search strategy for Embase.

**Appendix 3.**
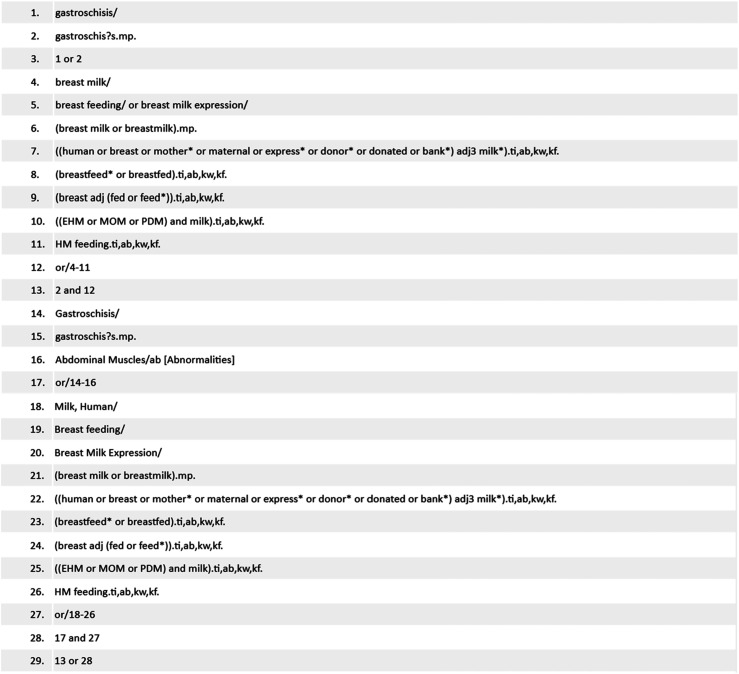
Search strategy for Cochrane Central Register of Randomized Controlled Trials.

**Appendix 4.**
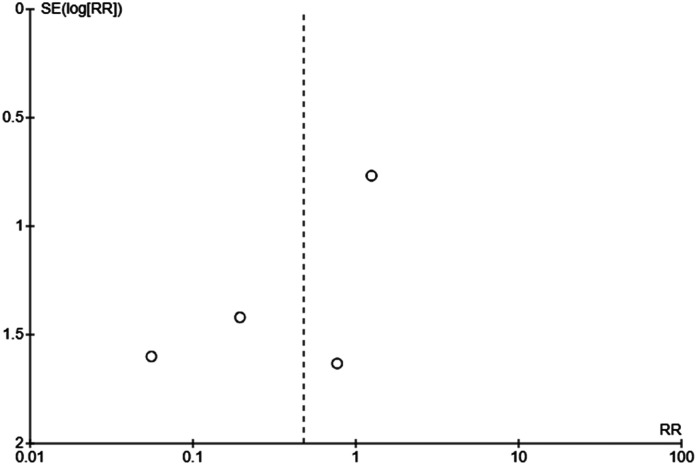
Funnel plot for studies which reported incidence of necrotizing enterocolitis.

**Appendix 5.**
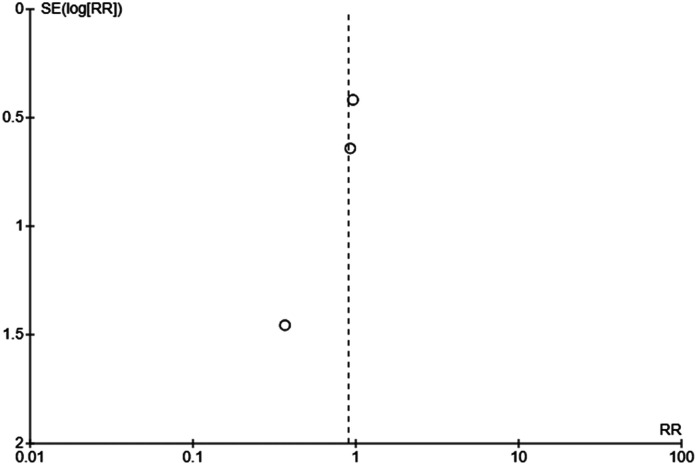
Funnel plot for studies which reported incidence of cholestasis.

**Appendix 6.**
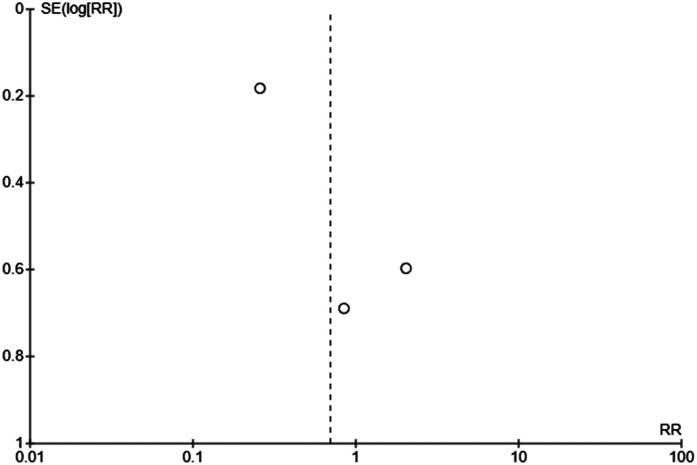
Funnel plot for studies which reported incidence of sepsis.

**Appendix 7.**
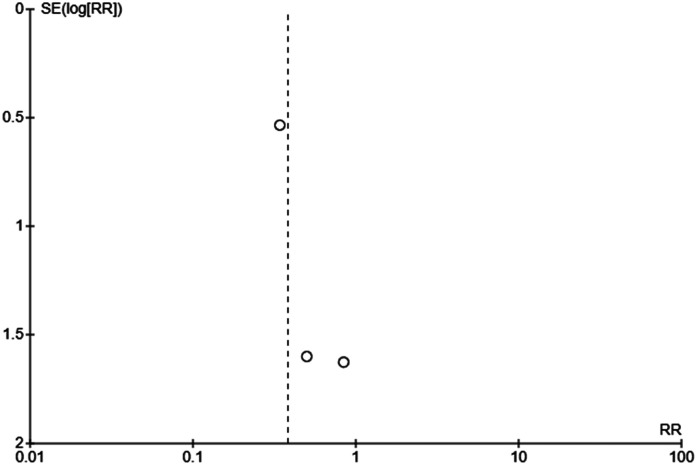
Funnel plot for studies which reported incidence of mortality.

## Appendix

### Abbreviations

GRADE: Grading of recommendations, assessment, development and evaluations
HR: Hazard ratio
NOS: Newcastle-Ottawa Scale
RR: Risk ratio.
